# Cervical Spinal Cord Injury During Prone Position Ventilation in the COVID-19 Pandemic

**DOI:** 10.7759/cureus.18958

**Published:** 2021-10-21

**Authors:** Abdulrahman M AlMutiri, Samer Alsulaimani, Abdulrahman J Sabbagh, Khalid M Bajunaid, Wail A Tashkandi, Saleh S Baeesa

**Affiliations:** 1 Surgery, King Abdulaziz University Faculty of Medicine, Jeddah, SAU; 2 Surgery, King Abdulaziz University Hospital, Jeddah, SAU; 3 Surgery, University of Jeddah, Jeddah, SAU; 4 Critical Care Medicine, King Abdulaziz University Faculty of Medicine, Jeddah, SAU

**Keywords:** sars-cov-2 infection, opll, ankylosis spondylitis, ventilation, prone position, spinal cord injury, cervical spine fracture, covid-19

## Abstract

The prone positioning of patients experiencing acute respiratory distress syndrome (ARDS) due to coronavirus disease 2019 (COVID-19), caused by severe acute respiratory syndrome coronavirus 2 (SARS-CoV-2), has been proven effective in optimizing oxygenation and lung function. However, such patients may be at risk of developing complications due to the prolonged prone position in intensive critical care. A 45-year-old COVID-19 female, not known with cervical spine disease, presented with progressive severe COVID-19-related hypoxemia that required intensive care unit admission for pulmonary care. She was positioned prone and ventilated for several weeks. She developed a rapidly advanced decreased level of consciousness and flaccid quadriparesis. CT and MRI scans of the cervical spine revealed C4/C5 fracture-dislocation with spinal cord compression in asymptomatic ankylosing spondylitis and focal ossification of a posterior longitudinal ligament. In addition, the patient had severe ARDS-SARS-CoV-2 hemodynamic instability. Surgery was not performed due to her critical condition, and the patient died from multi-organ failure. Patients with underlying cervical spine disease or deformity can be subjected to hyperextension and develop fatigue (stress) spinal fracture, leading to spinal cord compression. To our knowledge, this is the first case of spontaneous cervical spine fracture dislocation in a COVID-19 patient after several weeks in prone position ventilation in ICU. Hence, our case report raises the awareness of the possibility of devastating spinal cord injuries in prone position ventilation during the COVID-19 pandemic and the need for early screening using plain X-rays of these patients for cervical spine disease.

## Introduction

Coronavirus disease 2019 (COVID-19), caused by the severe acute respiratory syndrome coronavirus 2 (SARS-CoV-2), is an illness that mainly affects the respiratory system and leads to acute hypoxemia and respiratory distress, requiring admission to intensive care units in 0.9% to 32% of cases [1-4]. Different intensive care measures are undertaken in such patients to improve respiration and optimize lung function, including invasive and non-invasive procedures [5,6]. Of these measures, placing the patient in a prone position has been proven safe and effective in treating hypoxemic respiratory failure among COVID-19 patients by optimizing oxygenation, reducing inflammation, and improving lung function [6-9]. Improvement of respiratory and oxygenation parameters post-positioning was evaluated through several studies that reported the practice of prone positioning in intubated and awake, non-intubated patients [2,10,11]. In moderate SARS-CoV-2, awake prone positioning is thought to be more practical in reducing time and resource consumption and harboring less risk of developing adverse events that may complicate such procedures [11]. However, several complications were encountered in extended prone position ventilation, and hence, to the best of our knowledge, we present the first report of a devastating cervical spinal cord injury in a patient with COVID-19 as a complication of prone position ventilation.

## Case presentation

A 45-year-old female, known to be healthy, presented to the emergency department in August 2020, during the COVID-19 pandemic, complaining of cough, runny nose, generalized fatigue associated with vomiting, and diarrhea of four-day duration. She had been recently in close contact with a COVID-19-infected family member. The patient had no history of significant back pain that required a clinic visit or radiological investigation during history taking, and there were no neurological symptoms. Her past medical history was unremarkable, and, mainly, there was no previous spine trauma or surgery.

**Figure 1:**
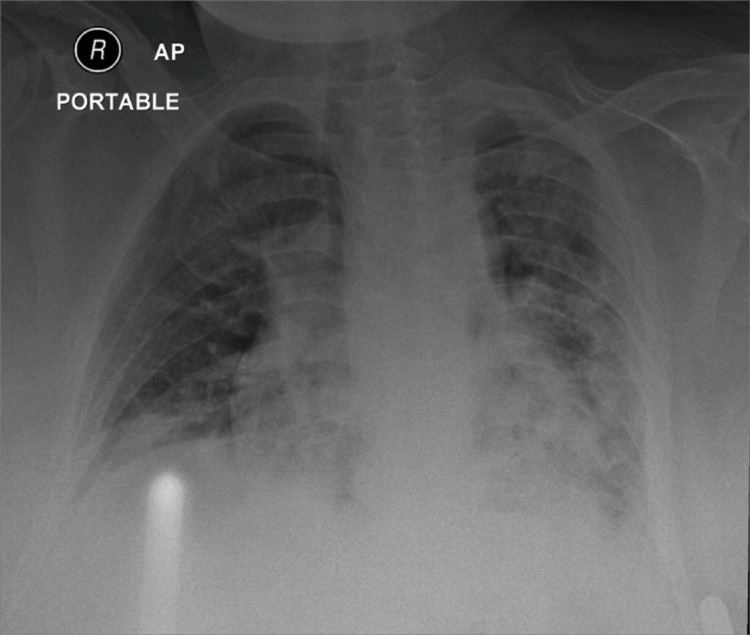
Chest X-ray revealing bilateral basal lung infiltration in the COVID-19 patient COVID-19, coronavirus disease 2019

On admission, the patient was conscious and alert. Her vital signs revealed a regular heart rate of 76 beats/min, blood pressure of 165/84 mmHg, normal respiration of 18/min with oxygen saturation (SpO_2_) of 95% on room air, and moderate fever of 38.8°C. General physical examination revealed an ill patient with a short neck and painless limited range of motion. There was no spine or large joint tenderness or deformity. A neurological exam revealed unremarkable cognitive, cranial nerves, and motor functions, and the patient could mobilize out of bed independently. The patient was admitted to the emergency room COVID-19 isolation facility for observation, and her polymerase chain reaction (PCR) test for SARS-CoV-2 through an oropharyngeal swab was positive conﬁrming COVID-19 infection. Initial laboratory studies on admission were unremarkable for renal, liver, and coagulation profiles apart from mild leukocytosis (13,420 per mm^3^) with lymphopenia (1500 per mm^3^). In addition, there was mild bilateral patchy pulmonary infiltration on the initial chest X-ray (Figure 1). The patient was admitted to the COVID-19 isolation ward for observation with O_2_ support via a mask and started on subcutaneous injection of enoxaparin 60 mg/0.6 mL daily and intravenous injection of dexamethasone 4 mg every eight hours.

After 48 hours from admission, the patient had progressive shortness of breath with hypoxia (SpO_2_ less than 90%) despite increasing O_2_ mask support and chest physiotherapy. The patient was transferred to the intensive care unit (ICU) due to worsening respiratory function diagnosed clinically and on chest X-ray from severe acute respiratory distress syndrome (ARDS) SARS-CoV-2 (ARDS-SARS-CoV-2). She was managed with awake non-invasive ventilation (NIV) in a prone position with a high-flow nasal cannula (HFNC) with significant improvement in oxygenation parameters (ratio of partial pressure of arterial oxygen [PaO_2_] to the fraction of inspired oxygen [FiO_2_] or PaO_2_/FiO_2_, and SpO_2_) and respiratory rate and started with empirical antibiotics. However, three days later, her COVID-19-related hypoxemia worsened, and the patient was required to be sedated and intubated and managed in prone position ventilation with adequate oxygenation parameters. The patient was nursed in a prone potion with the head of the bed elevated 20°-30° for a period varying from 9 hours every day to up to 72 hours and required three to four nurses for turning her in bed. She was turned supine when FiO_2_ dropped to less than 60% with positive end-expiratory pressure less than 10 cm H_2_O for more than four hours.

In the third week of ICU admission, the patient developed decreased urine output with increased creatinine levels and subsequent acute renal injury, requiring vasopressors and hemodialysis. She also developed sepsis from pneumonia and urinary tract infection due to *Pseudomonas aeruginosa* and *Klebsiella pneumoniae*, respectively, which responded to intravenous antibiotics (azithromycin and meropenem) for two weeks. In addition, she received the remdesivir intravenous infusion of 100 mg daily for seven days. During these complications, her conscious level was adequate, and she was communicating with nurses with movements of all extremities.

**Figure 2:**
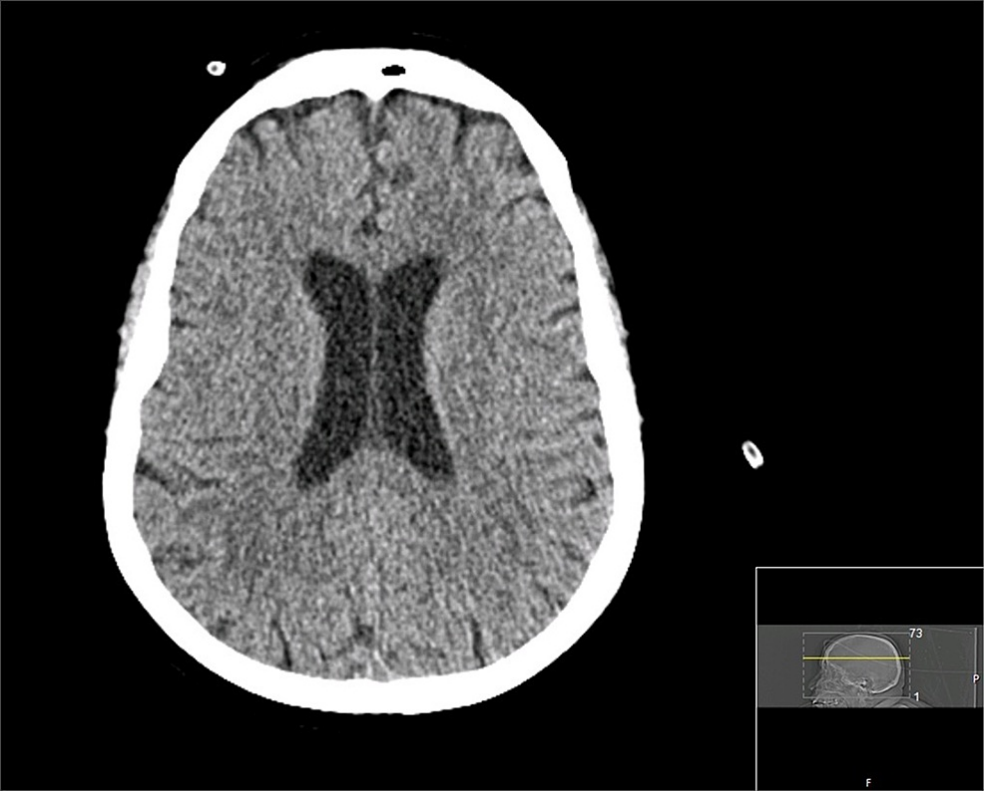
Axial brain CT scan revealing no cerebral insult

**Figure 3:**
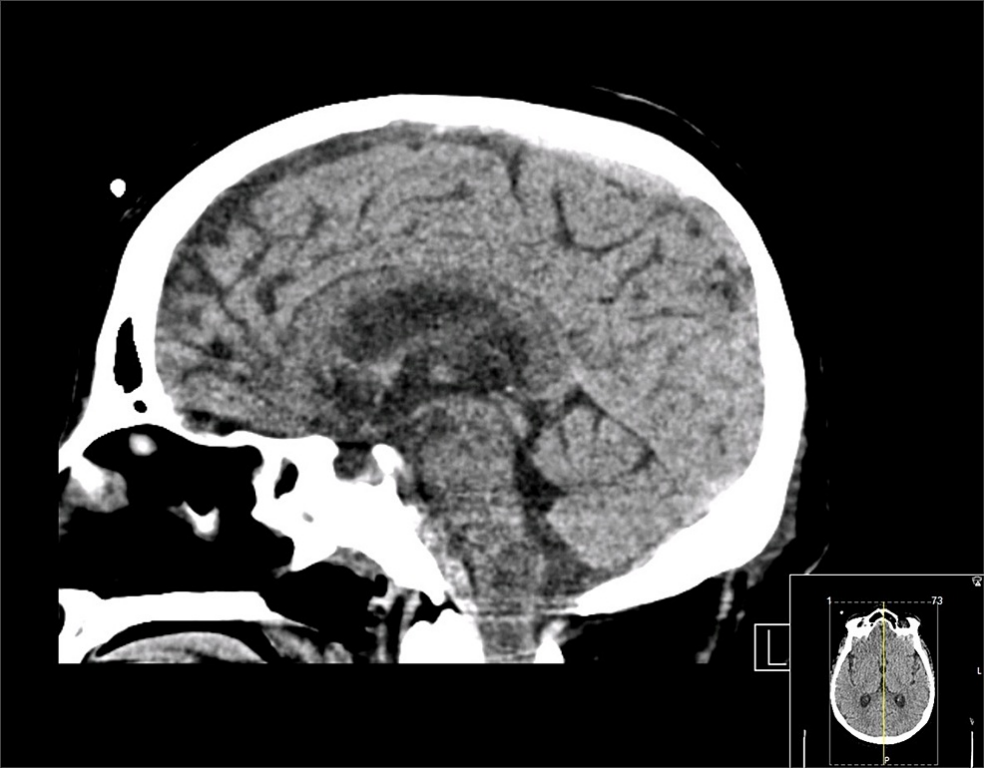
Sagittal brain CT scan revealing hypodensity of the lower brain stem

In the fifth week, while in a prone position, the patient developed episodes of bradycardia and hypotension and became less responsive with a decreased level of consciousness and flaccid extremities. A stroke was suspected, and she was turned supine and resuscitated and planned for urgent imaging. Urgent percutaneous tracheostomy was performed to replace the MRI-incompatible endotracheal tube. Urgent brain and cervical spine CT revealed no evidence of stroke; there was a hypodensity at the lower brain stem (Figures 2, 3).

**Figure 4:**
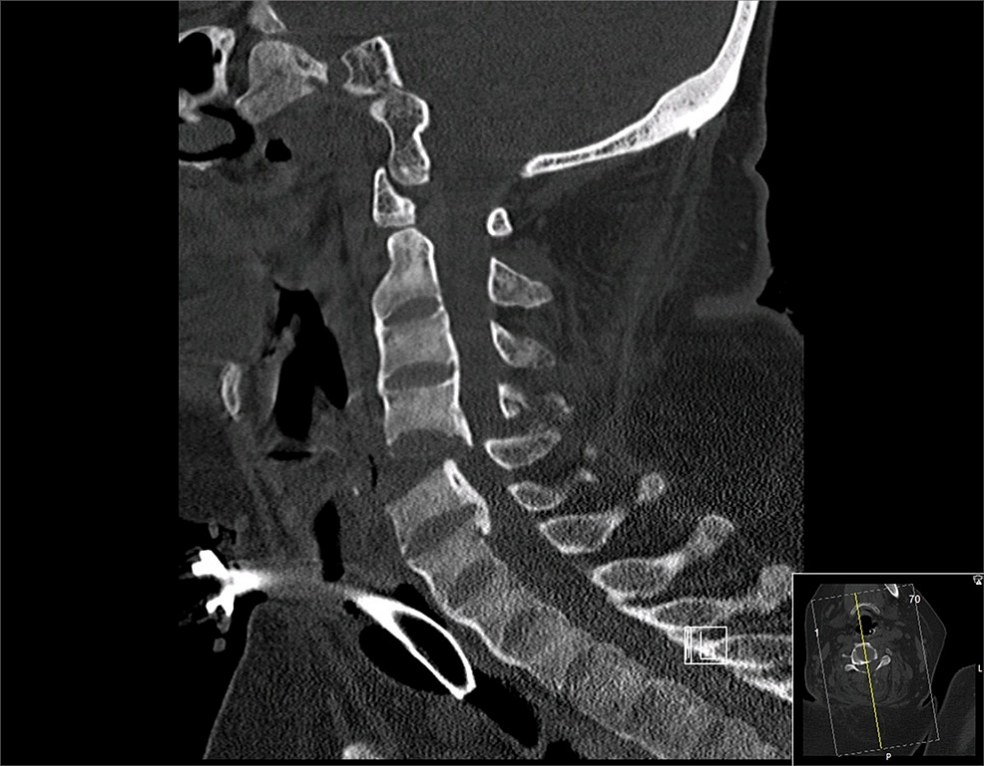
Sagittal cervical CT scan revealing fracture-dislocation at C4-5 and underlying evidence of ankylosing spondylitis and focal ossification of the posterior longitudinal ligament

**Figure 5:**
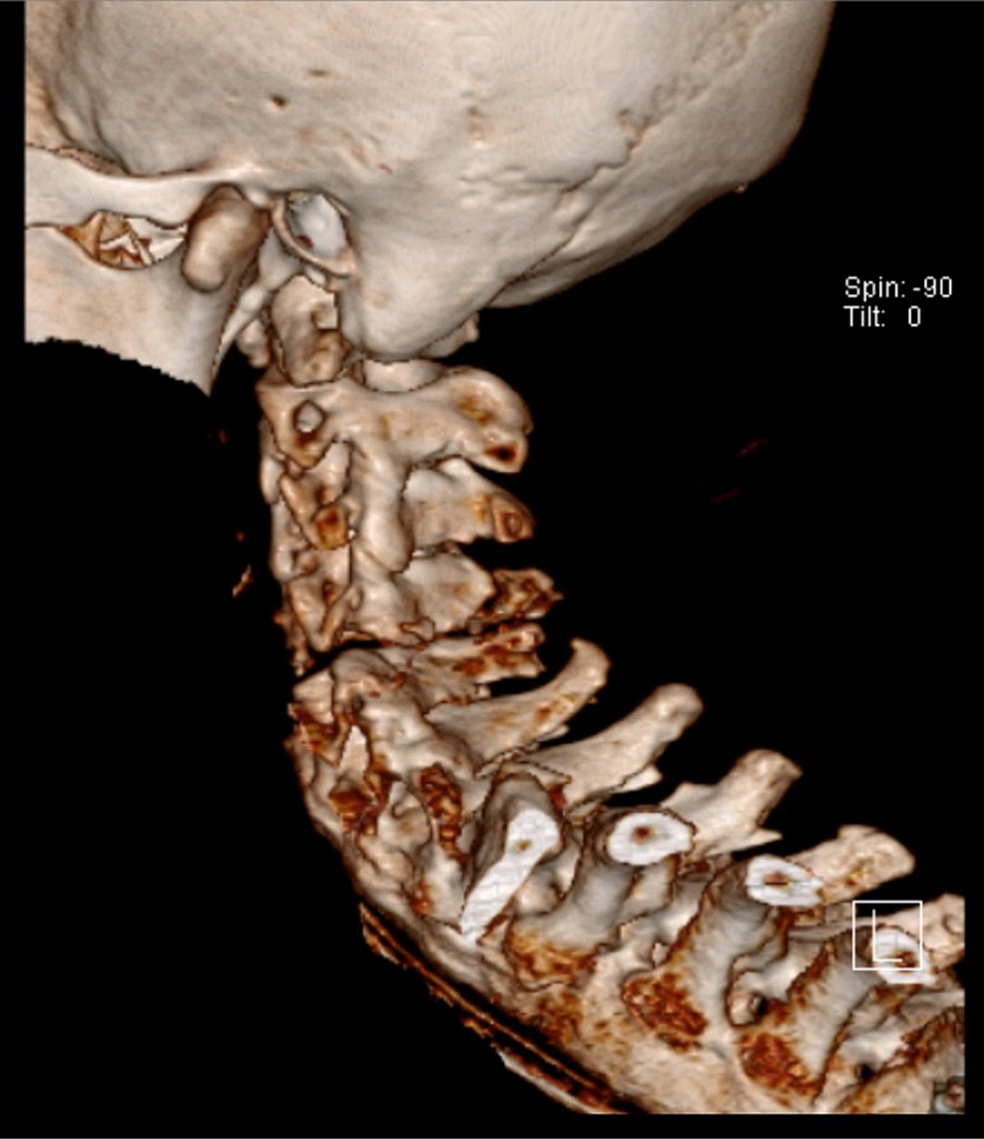
Cervical CT scan with 3D reconstruction revealing fracture-dislocation at C4-5

While on the CT table, cervical spine CT was performed and revealed a fracture-dislocation at the C4-5 level with retrolisthesis and underlying features of ankylosing spondylitis (AS) in addition to the focal ossification of a posterior longitudinal ligament (OPLL) at the same level (Figures 4, 5).

**Figure 6:**
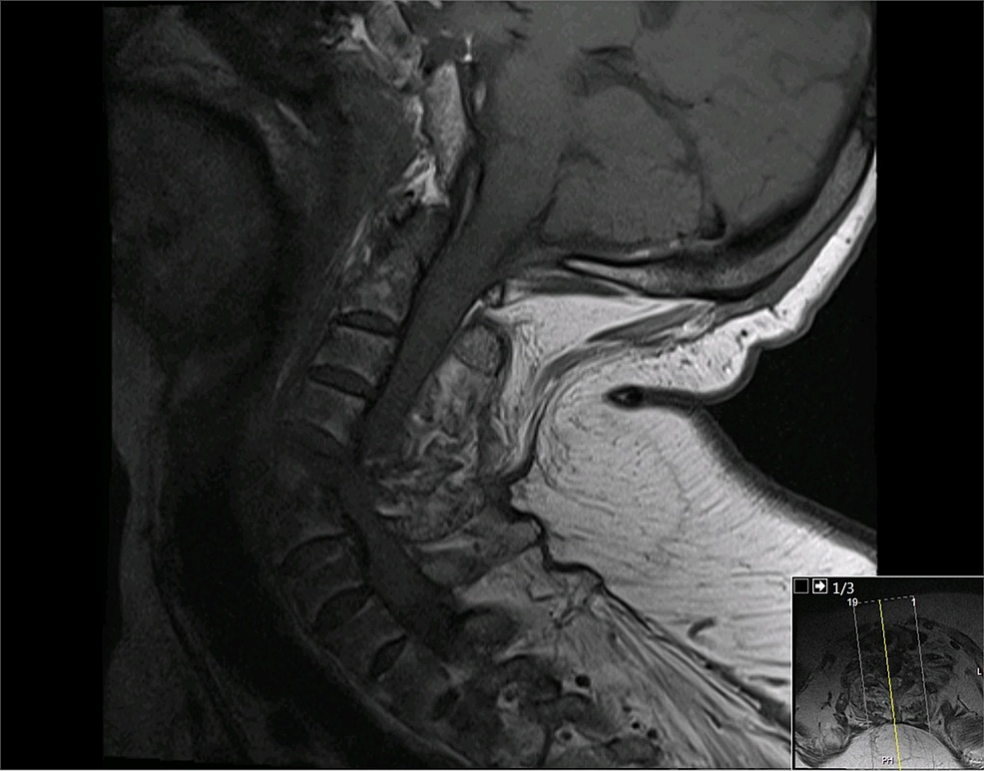
Cervical spine MRI in the sagittal T1-weighted sequence revealing a disruption injury at the C4-5 disk site and spinal cord compression

**Figure 7:**
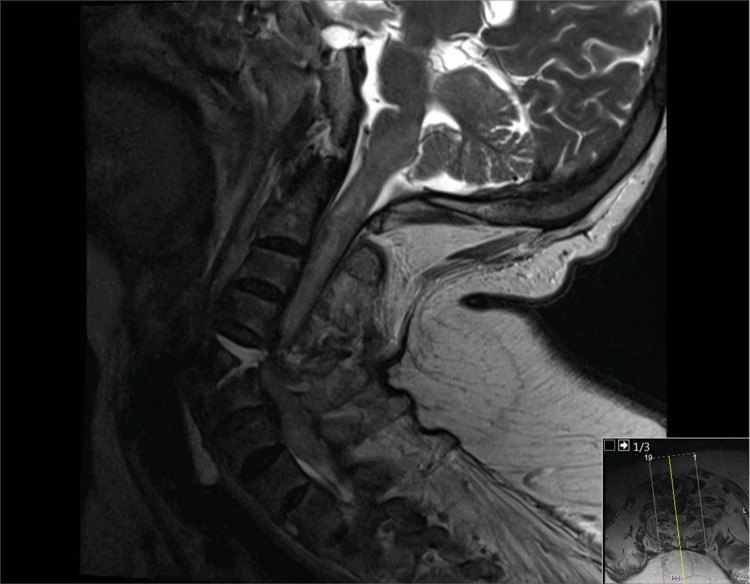
Cervical spine MRI in the sagittal T2-weighted sequence revealing a disruption injury at the C4-5 disk site and spinal cord compression. In addition, there is extensive spinal cord edema extending to the brain stem

**Figure 8:**
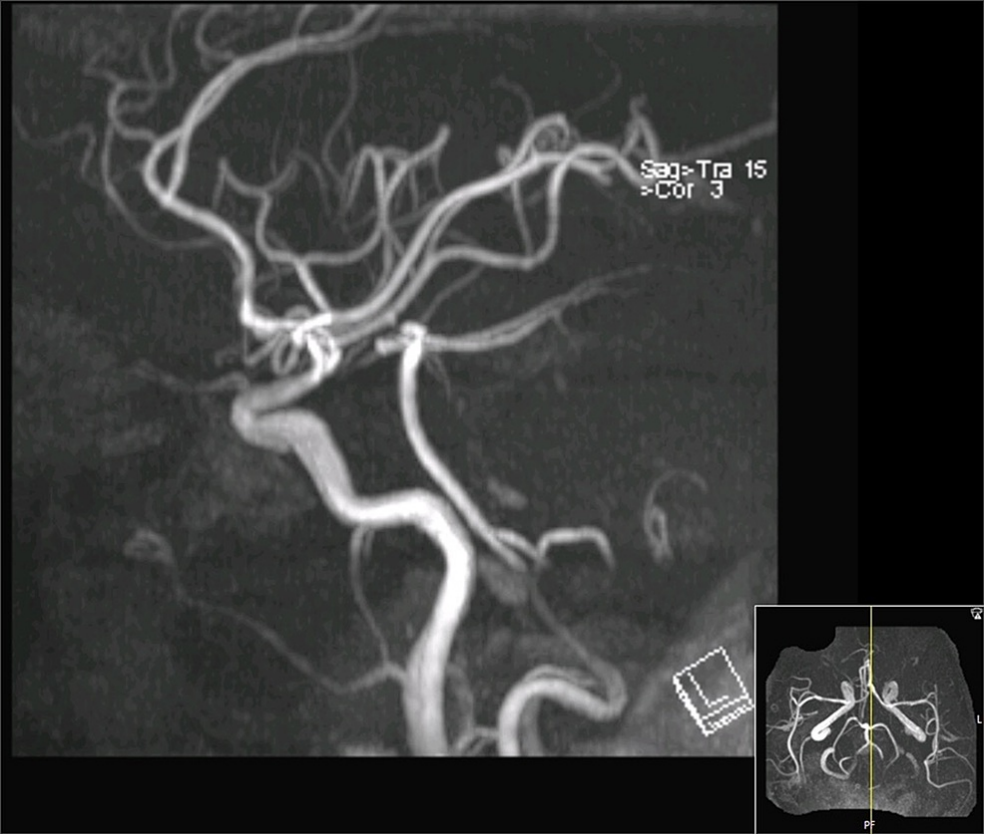
MR angiography scan revealing adequate filling of vertebral vessels

Subsequently, brain and cervical spine MRI was performed, which revealed no ischemic brain insult. Instead, there was cervical spinal cord edema extending to the lower brain stem and significant narrowing of the spinal canal and spinal cord compression. The fractured cervical spine at the C4-5 level was associated with a small epidural hematoma (Figures 6, 7). MR angiography did not demonstrate any compromise of the carotid or vertebral vessels (Figure 8).

**Figure 9:**
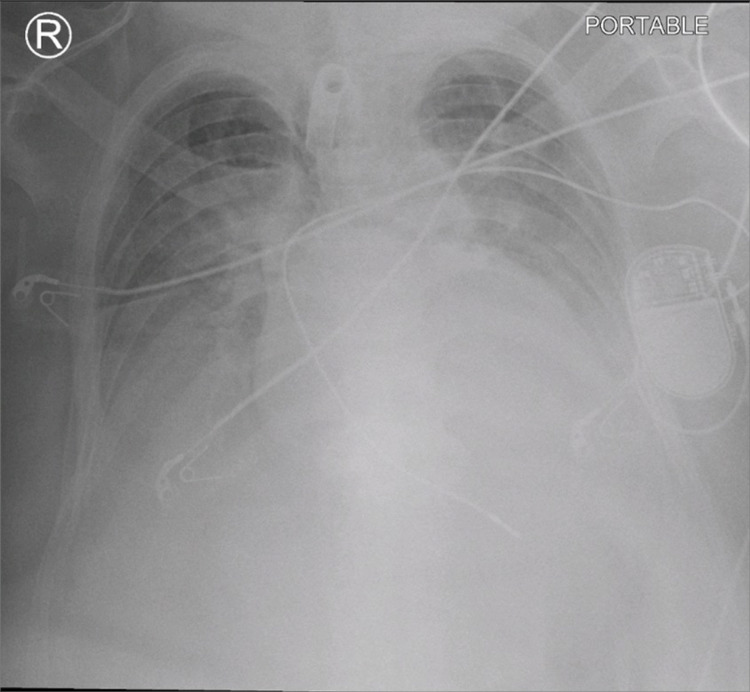
Chest X-ray revealing severe acute respiratory distress syndrome A pacemaker was inserted for arrhythmia.

The patient was managed with ventilation in supine with adequate cervical spine mobilization of alignment with gentile traction. However, she developed several episodes of bradycardia and hypotension and developed hemodynamic instability due to atrial fibrillation, which required an emergency insertion of a cardiac pacemaker. In addition, her chest X-rays and ventilation parameters continued to worsen due to severe ARDS-SARS-CoV-2 (Figure 9).

Due to her severe illness, posterior cervical spine fixation could not be performed, and the patient developed multi-organ failure. She developed ventricular fibrillation followed by asystole, which failed to respond to cardiopulmonary recusation, and the patient was declared dead after eight weeks from ICU admission.

## Discussion

Since the publication of the Proning Severe ARDS Patients (PROSEVA) trial in 2013, the prone positioning recommendation to help to improve respiratory function and oxygenation in critically ill patients with ARDS has extended its implementation during the COVID-19 pandemic [8]. However, during the worldwide extended prone position ventilation use in the COVID-19 pandemic, there have been rising concerns of its associated adverse events and complications that can be devastating, with their management hampered by the isolation precaution and personal protective equipment scarcity, and also by patient condition [12,13]. Several studies have reported certain complications of prone positioning in COVID-19 patients, including pressure-related skin and scalp ulcers, and facial and ocular injuries [14,15]. However, neurological complications related to prone position were reported as brachial plexus and peripheral nerve compression injuries [16-19]. Therefore, we performed a literature review using MEDLINE and PubMed databases to identify articles published between 2013, since the recommendation of prone position ventilation, and July 2021, using the following keywords: “ankylosing spondylitis,” “COVID-19,” “spinal cord injury,” and/or “fracture.” Our search did not reveal any previously reported similar cases.

Herein we present the first case of a devastating spinal cord injury case due to cervical spine fracture-dislocation in a 45-year-old female COVID-19 patient during mechanical ventilation in a prone position. The patient had no history of neck trauma or previously diagnosed spine disease. However, her cervical CT scan revealed C4-5 fracture-dislocation in the underlying key hallmark radiological findings of AS and OPLL. The exact mechanism underlying the spinal injury is uncertain; however, we believe it occurred secondarily to persistent neck hyperextension with rotation posture during prone positioning, which is the primary mechanism of cervical spine injury in 75% of patients with AS [20]. In addition, extended prone posture may result in building stress or fatigue on the calcified ligaments and disks of the cervical spine and subsequent fracture, which is further aggravated by underlying osteoporosis.

On the other hand, as high as 42% of patients with AS present with cervical injuries that are either missed or delayed in diagnosis [20]. Neck stabilization and low weight traction in critically ill patients for such unstable injury is challenging. Our patient was initially treated supine with careful cervical realignment, positioning, and minimal weight traction to reduce and stabilize the cervical spine's fracture-dislocation. Unfortunately, her neurological deficit did not improve, and she was severely ill and hemodynamically unstable to undergo posterior spinal cord decompression and cervical fixation.

We emphasize, through presenting this rare complication due to prone positioning ventilation, the need for the earlier identification of COVID-19 patients with a known history of previous spinal trauma or surgery and those with degenerative spine disease and deformity, particularly, radiculopathy or myelopathy. Since a decrease in prone positioning time is not always feasible in critically ill COVID-19 patients and limited patient-staff interaction, assessments by trained ICU staff before and during prone should be undertaken and documented. Also, alternate patient turning is advised, and frequently switching from prone to sides may avoid pronged neck rotation and extension, reducing the risk of cervical spine injury, particularly, in elderly and high-risk patients. Frequent clinical evaluation in awake patients to evaluate associated neck pain, and change in neurological examination of the extremities during prone or a change in position should be practiced. Early cervical spine screening using X-rays, CT, or MRI, if indicated, before admission to ICU for prone positioning ventilation, which our hospital mortality and morbidity committee recommended, should be performed for COVID-19 patients with suspected cervical spine disease.

## Conclusions

Although prolonged prone position ventilation is feasible, relatively safe, and widely implicated in treating critically ill COVID-19 patients and SARS-CoV-2, devastating neurological complications from spinal cord injury can occur. To the best of our knowledge, we have described the ﬁrst case in the literature of spinal cord injury from cervical fracture-dislocation in a patient with underlying asymptomatic cervical spine AS/OPLL disease during mechanical ventilation in a prone position. In addition, we highlighted the need to increase the awareness among ICU care providers in prompt identiﬁcation and early screening, using spine X-rays, of at-risk patients with diagnostic uncertainty of cervical spine disease prior to prone position ventilation to prevent such a devastating complication.
